# A randomized, double-blind, active-controlled, double-dummy, parallel-group study to determine the safety and efficacy of oxycodone/naloxone prolonged-release tablets in patients with moderate/severe, chronic cancer pain

**DOI:** 10.1177/0269216311418869

**Published:** 2012-01

**Authors:** Sam H Ahmedzai, Friedemann Nauck, Gil Bar-Sela, Björn Bosse, Petra Leyendecker, Michael Hopp

**Affiliations:** School of Medicine and Biomedical Sciences, University of Sheffield, UK; Department of Palliative Medicine, University of Göttingen, Germany; Division of Oncology, Rambam HCC, Technion-Israel Institute of Technology, Haifa, Israel; Mundipharma Research GmbH & Co. KG, Limburg, Germany; Mundipharma Research GmbH & Co. KG, Limburg, Germany; Mundipharma Research GmbH & Co. KG, Limburg, Germany

**Keywords:** Analgesia, constipation, naloxone, neoplasms, oxycodone, pain

## Abstract

**Objective:** An examination of whether oxycodone/naloxone prolonged-release tablets (OXN PR) can improve constipation and maintain analgesia, compared with oxycodone prolonged-release tablets (OxyPR) in patients with moderate/severe cancer pain.

**Methods:** Randomized, double-blind, active-controlled, double-dummy, parallel-group study in which 185 patients were randomized to receive up to 120 mg/day of OXN PR or OxyPR over 4 weeks. Efficacy assessments included Bowel Function Index (BFI), Brief Pain Inventory Short-Form (BPI-SF), laxative and rescue medication use. Quality of life (QoL) and safety assessments were conducted.

**Results:** After 4 weeks, mean BFI score was significantly lower with OXN PR; mean total laxative intake was 20% lower with OXN PR. Mean BPI-SF scores were similar for both treatments and the average rate of analgesic rescue medication use was low and comparable. QoL assessments were stable and comparable with greater improvements in constipation-specific QoL assessments with OXN PR. Overall, rates of adverse drug reactions were similar.

**Conclusions:** OXN PR provides superior bowel function in cancer pain patients, compared with OxyPR, without compromising analgesic efficacy or safety. This study confirms that OXN PR is well tolerated and efficacious in cancer pain patients and results are in line with those seen in non-malignant pain patients.

## Introduction

Patients with cancer experience a wide variety of problems related not only to the disease itself, but often to the treatments involved and their related side effects. Pain is a particular concern for cancer patients, having a significant impact on quality of life.^1,2^ More than 80% of cancer patients with advanced metastatic disease experience pain caused largely by direct tumour infiltration.^2^ Opioids are recommended for the management of moderate/severe cancer pain by the WHO and current guidelines,^1–4^ and are recognized as the treatment of choice.^2^ The semi-synthetic opioid analgesic oxycodone has become a cornerstone of pain management in a wide range of settings,^5^ including cancer-related pain.^6,7^ However, successful management of pain with opioids requires that the benefits of these agents outweigh the impact of treatment-related side effects such as constipation.^8,9^ Up to 95% of patients with cancer experience constipation, with highest incidence observed in those who receive opioid therapy.^10^ This complication can cause further deterioration in the quality of life of patients with cancer.

The effects of opioids are determined by the location of opioid receptors. Activation of opioid receptors in the central nervous system results in analgesia, but activation of opioid receptors in the gut wall leads to reduced gut motility, delayed gastric emptying, increased sphincter tone and slower gut transit time.^11^ Consequently, patients can experience constipation, gastro-oesophageal reflux, abdominal cramping, spasm and bloating; collectively these symptoms are known as opioid-induced bowel dysfunction.^12,13^ Opioid-induced constipation (OIC) is the most frequently reported and persistent adverse event (AE) in patients receiving opioid analgesia.^14^

Current management strategies for OIC are non-specific, often ineffective and are largely lacking a good evidence base.^15^ Laxatives are frequently used and can be effective, although many patients still do not achieve adequate symptom relief, as they fail to address the underlying opioid-related mechanisms.^16,17^ Use of peripherally acting opioid antagonists has been identified as a promising approach; these agents specifically target gastrointestinal (GI) receptors without limiting the central analgesic activity of opioids.^18,19^ Naloxone is a peripherally acting opioid antagonist with low systemic bioavailability (<3%) following oral administration, due to extensive first-pass hepatic metabolism.^20–22^ Consequently, orally administered naloxone acts almost exclusively on opioid receptors in the GI tract.^20,23^ Targeting peripheral receptors whilst sparing central analgesic function through combining naloxone with oxycodone has emerged as a promising approach for managing OIC, and generated much academic and clinical interest.^16,17,24^

Three large, randomized, placebo-controlled Phase III trials in patients with non-cancer pain,^25–27^ plus a prospectively planned pooled analysis of two of these studies,^28^ have already confirmed analgesic efficacy of the combination of prolonged-release (PR) oxycodone/naloxone (OXN PR), while also demonstrating benefits in terms of bowel function versus oxycodone PR (OxyPR) alone. Importantly, OXN PR did not lead to a reduction in analgesic efficacy, compared with OxyPR. Moreover, high analgesic efficacy and a positive effect on bowel function associated with OXN PR have been shown in a long-term study,^29^ and in a large observational study.^30^ The trials described above have demonstrated the benefits of the combination of oxycodone with naloxone in patients with chronic non-cancer pain. Because opioid drugs are recommended for the management of moderate/severe pain in cancer patients,^1–4,31^ the aim of the current study was to investigate whether OXN PR can also improve constipation and maintain analgesia, compared with OxyPR, in the cancer pain patient population.

## Methods

### Study design

The OXN2001 trial (ClinicalTrials.gov identifier NCT00513656)^32^ was a 4-week, international, multicentre, randomized, double-blind, active-controlled, double-dummy, parallel-group, Phase II study, designed to evaluate the safety and efficacy of OXN PR in patients with moderate/severe chronic cancer pain. The study was initiated on 2 November 2007 and the extension phase was completed on 17 August 2010. Following the screening period (3–10 days before randomization), eligible patients stopped their pre-study opioid and laxative medication and were randomized (on Day 1/Visit 2) to switch directly to either OXN PR or OxyPR during the 4-week double-blind treatment phase. During the core phase of the study, all subjects, investigators and sponsor personnel were blinded. Treatments were masked in a double-dummy fashion, whereby subjects randomized to receive OXN PR were given OXN PR and OxyPR placebo, and subjects randomized to receive OxyPR were given OxyPR and OXN PR placebo. Patients attended three further clinic visits (on Days 8, 15 and 29), and received four additional telephone calls (on Days 2, 4, 6 and 22). Patients could enter an open-label extension phase, the details and results of which will be reported separately. The study was performed in full compliance with applicable Good Clinical Practice^33^ and regulations, and in accordance with the Declaration of Helsinki.^34^

There were two primary objectives: (i) to determine whether patients with moderate/severe cancer pain taking OXN PR experience an improvement in symptoms of constipation, as measured by the validated Bowel Function Index (BFI),^35–37^ compared with patients taking OxyPR alone; and (ii) to compare efficacy for management of chronic cancer pain, as assessed by the Brief Pain Inventory–Short Form (BPI-SF).^38^ The secondary objectives included a comparison of effects on laxative and rescue medication use, quality of life (QoL) and safety. Regardless of treatment group, patients were titrated up to a maximum of 120 mg/day oxycodone PR (if required). Open-label oxycodone immediate-release capsules (OxyIR) were available to patients as rescue medication, up to a maximum of six doses per 24 h. Patients who needed to titrate up to oxycodone PR 120 mg/day and who regularly required two or more rescue doses of OxyIR were withdrawn from the study. Bisacodyl tablets were available as laxative rescue medication, up to a maximum of five doses within seven consecutive days.

### Study population

Eligible patients were aged ≥ 18 years, with a diagnosis of cancer and a documented history of moderate/severe, chronic cancer pain, requiring round-the-clock opioid therapy (equivalent to OxyPR 20–80 mg/day at the start of the trial). Subjects had to be willing and able (e.g. mental and physical condition) to participate in all aspects of the study, including use of medication, completion of subjective evaluations, attending scheduled clinic visits, completing telephone contacts, and compliance with protocol requirements as evidenced by providing written, informed consent. Patients were excluded from the study for the following reasons: evidence of clinically unstable disease or significant cardiovascular, renal, hepatic or psychiatric disease; clinically significant GI disease, or significant structural abnormalities of the GI tract; cyclic chemotherapy within 2 weeks before screening visit or planned during the core study (shown in the past to influence bowel function); radiotherapy that would influence bowel function or pain during the double-blind phase.

### Study assessments

Evaluations of bowel function (BFI),^35–37^ pain control (BPI-SF),^39^ use of rescue medication and use of laxative medication during the last 7 days were performed at each clinic visit. The co-primary efficacy variable was average pain over the last 24 h based on BPI-SF (non-inferiority bound 1.0). Co-primary efficacy assessments were based on changes from baseline after 4 weeks of treatment.

Quality-of-life assessments, including the European QoL (EuroQoL) EQ-5D instrument^40^ and European Organization for Research and Treatment of Cancer QoL Questionnaire-Core 30 (EORTC QLQ-C30)^41,42^ were conducted at screening and study end. In addition, Patient Assessment of Constipation Symptoms (PAC-SYM) was conducted at screening, randomization and 4 weeks.^43^ Adverse events were noted at every assessment post-randomization. In addition, vital signs, clinical laboratory tests, physical examinations and 12-lead electrocardiographs (ECGs) were conducted throughout the study. Patients were followed up regarding AEs and adverse drug reactions (ADRs) after study completion. At randomization, Days 2, 4, 6 and 8, patients were asked about symptoms of opiate withdrawal (using the modified Subjective Opiate Withdrawal Scale [SOWS]).^27,44^

### Randomization and sample size calculation

Patients were assigned to treatments (1:1 allocation ratio) using a pseudo-random number generator in a computer program. During visit 2, eligible subjects were randomized to one of two treatment groups (i.e. OXN PR or OxyPR) according to a randomization schedule prepared by the Clinical Supplies Department of the Sponsor or an associated company. Treatment assignments were randomized within blocks of fixed size. No stratification was done. The study was designed to have a power of 80% to detect a treatment difference of 12 on the BFI on a two-sided level of significance of *α* = 0.05 assuming a common standard deviation (SD) of 26.

Non-inferiority of OXN PR to OxyPR regarding pain intensity could be concluded on a one-sided level of significance of *α* = 0.05 assuming a non-inferiority bound of 1.0 and a common SD of 2.0.

### Statistical analysis

For the primary analyses, analysis of covariance (ANCOVA) was used to compare treatments regarding primary and co-primary endpoints at 4 weeks, adjusting for baseline observation, and using the last observation carried forward (LOCF) approach for missing values. For the BFI, the null hypothesis was a zero difference (on average) between treatment groups at the final visit. The alternative hypothesis was that there is a difference between the treatment groups. Two-sided tests were performed at the 5% significance level. For BPI-SF, the null hypothesis was a difference of −1 (on average) between treatment groups at the final visit, in favour of Oxy PR (OXN PR inferior to OxyPR). The alternative hypothesis was a difference greater than −1 (OXN PR non-inferior to Oxy PR). One-sided *t*-tests were performed at the 5% significance level.

Additional sensitivity analyses were performed for co-primary endpoints using the same ANCOVA analyses on the non-LOCF data and baseline observation carried forward (BOCF) data. Mixed-effects models for repeated measures (MMRM) analyses were also conducted, adjusting for visit and treatment × visit interaction, and assuming a constant treatment effect over visits in respective MMRM analyses. For other efficacy outcomes and safety data, summary statistics were produced.

## Results

### Patient disposition

**Figure 1.**
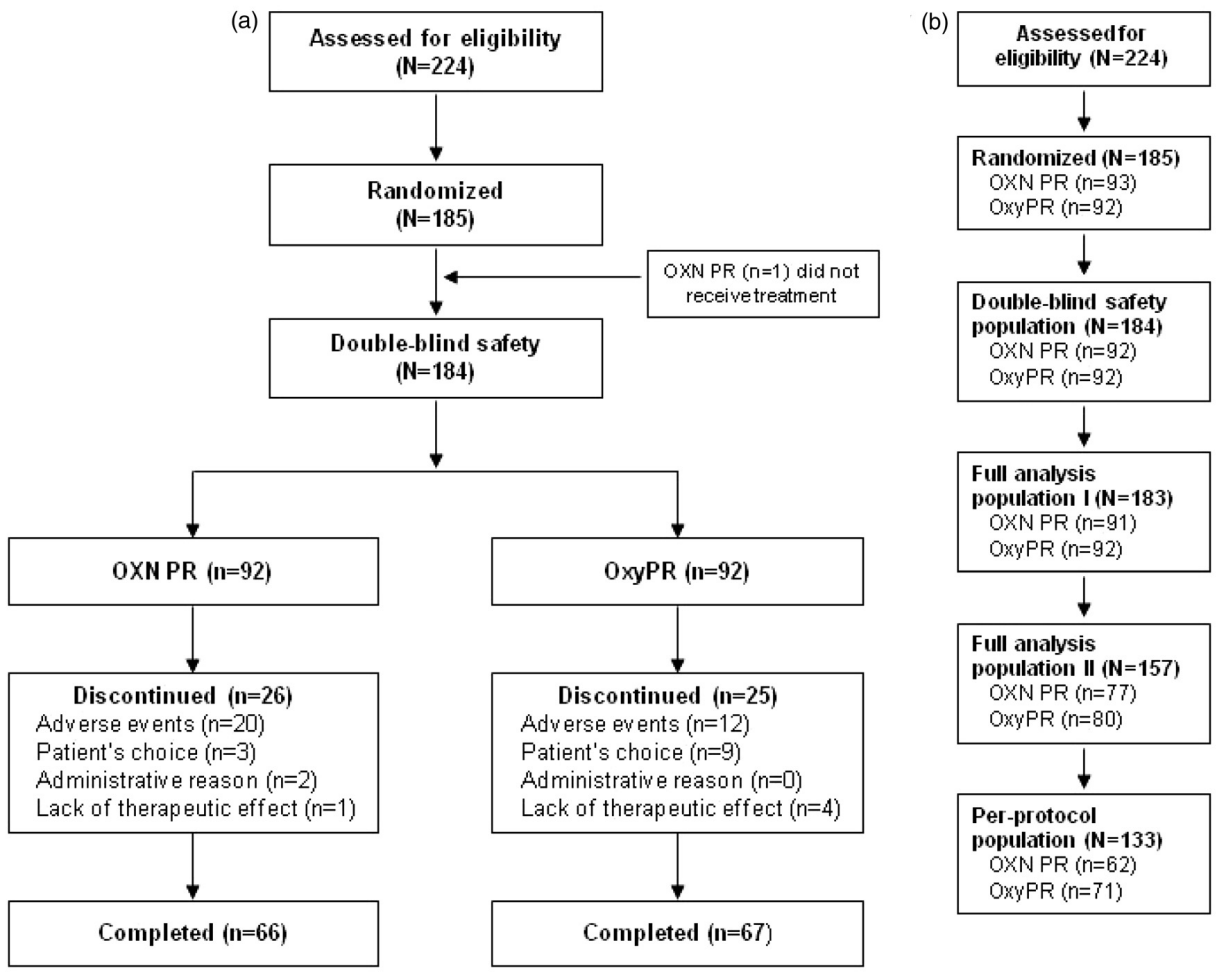
(a) Patient disposition; (b) study populations. The double-blind safety population included all patients who received any dose of study medication; the full-analysis population I excluded one patient from the double-blind safety population who did not receive treatment and was hence not included in the analysis; the full-analysis population II excluded those patients from the original full-analysis population who discontinued due to AEs other than constipation or lack of efficacy within the first 14 days (in order to obtain reliable BFI values and prevent possible skewing due to these early withdrawals); the per-protocol population included patients who received at least one dose of study medication during the double-blind phase and who sufficiently complied with the study protocol. OXN PR: oxycodone/naloxone prolonged-release tablets, OxyPR: oxycodone prolonged-release tablets, AE: adverse event, BFI: Bowel Function Index.

Of 224 enrolled patients, 185 were randomized, 184 were part of the double-blind safety population and 183 were included in the full analysis population I; the full analysis population II consisted of 157 patients and the per-protocol population of 133 patients. During the blinded data evaluation it was recognized that 28 patients randomized dropped out early but not due to AE constipation and not due to lack of effect. It was decided at a blinded stage to exclude those subjects from the full analysis population I as those patients would not give valid information about the drug effects regarding BFI if the distribution was not equal in both treatments arms. The primary analysis (superiority testing) of BFI was performed in an intention-to-treat manner on the full analysis II population. The patient dispositions and study populations are shown in Figure 1A and B. Overall, 133/184 (72.3%) patients completed the study. Rates of discontinuation were similar for OXN PR (26/92 [28.3%]) and OxyPR (25/92 [27.2%]). In both groups, the primary reason for discontinuation was AEs (OXN PR, *n* = 20; OxyPR, *n* = 12).

### Patient demographics and baseline characteristics

Demographic characteristics were well balanced between treatment groups. The only exception to this was a slightly higher percentage of patients aged ≤ 65 years in the OXN PR group, compared with the OxyPR group (65.2 vs. 48.9%, respectively; Table 1). At the start of the study, 183/184 (99.5%) patients suffered from constipation induced or worsened by their opioid medication (OXN PR 92/92 [100%]; OxyPR 91/92 [98.9%], the one subject in the OxyPR group had constipation in their medical history but it was not recorded as ongoing at visit 1; Table 1). A similar number of subjects in each treatment group took laxatives (96.7% in the OxyPR group and 95.7% in the OXN PR group). The most frequently taken laxatives were lactulose, bisacodyl and sennoside. At baseline, the most frequently reported primary cancer sites were breast (19%), lung (13%) and prostate (10%). Twenty-six per cent of patients had bone metastases. Most frequently used pre-study opioids were fentanyl (29%), morphine (79%), oxycodone (85%) and tramadol (35%).

**Table 1.** Patient demographics and clinical characteristics (double-blind safety population)

	OXN PR	OxyPR
Variable	*n* = 92	*n* = 92
Age, years		
Mean (SD)	61.86 (10.93)	64.30 (9.63)
Median (range)	62.0 (36–84)	66.0 (42–82)
Age group, *n* (%)		
≤65 years	60 (65.2)	45 (48.9)
>65 years	32 (34.8)	47 (51.1)
Sex, *n* (%)		
Male	48 (52.2)	46 (50.0)
Female	44 (47.8)	46 (50.0)
Race, *n* (%)		
Caucasian	92 (100.0)	91 (98.9)
Black	0 (0)	1 (1.1)
Constipation induced or worsened by opioid medication, *n* (%)	92 (100.0)	91 (98.9)
	*n* = 90	*n* = 90
Body mass index, kg/m^2^		
Mean (SD)	25.34 (5.75)	25.62 (5.13)
Median (range)	24.7 (15–39)	25.6 (16–41)

SD: standard deviation, OXN PR: oxycodone/naloxone prolonged-release tablets, OxyPR: oxycodone prolonged-release tablets.

### Exposure to study medication

The majority of patients in the OXN PR and OxyPR groups received study medication for ≥ 4 weeks (59.8 vs. 67.4%, respectively), and had similar mean (SD) durations of study participation (23.58 [9.54] vs. 25.05 [8.37] days, respectively) and daily doses (46.59 [22.58] vs. 43.09 [19.31] mg/day, respectively).

### Efficacy

#### Primary endpoints

Perception of constipation varies from patient to patient and results from three randomized controlled trials have shown that the BFI is a validated, easy-to-use questionnaire for the measurement of opioid induced constipation.^35^ It is a three-item questionnaire that takes into account subjective criteria often reported by the patient. Using analysis of standard error of measurements and one-half SD characteristics of each BFI component, Rentz et al. reported that changes in BFI score ≥12 points represent clinically meaningful changes while score changes of less than 7.5 points are unlikely to be clinically meaningful in patient’s perception of their bowel habits.^35^ At randomization, mean (SD) BFI values were high and comparable in the OXN PR and OxyPR groups (63.97 [17.42] vs. 62.40 [23.56], respectively), and similar to baseline assessments in previous OXN PR Phase III trials,^25–27^ indicating that patients suffered from constipation caused or aggravated by opioid medication. The difference in change from baseline in BFI score (ΔBFI) between groups was statistically significant (LOCF, ΔBFI = −11.14; 95% confidence interval [CI]: −19.03 to −3.24; *p* < 0.01) These findings were underlined by the results of MMRM (with treatment by visit interaction, ΔBFI = −10.8; 95% CI: −18.8 to −2.8; *p* = 0.018), MMRM (assuming a constant treatment effect, ΔBFI = −12.36; 95% CI: −19.05 to −5.67; *p* < 0.01), BOCF (ΔBFI = −10.85; 95% CI: −18.63 to −3.073; *p* < 0.01 and LOCF analyses (per-protocol population: ΔBFI = −14.78; 95% CI: −23.03 to −6.53; *p* < 0.01). A statistically significant difference between treatments in favour of OXN PR was observed already at week 1. Taking all analyses into account, OXN PR is both statistically significantly superior in respect to BFI and has also demonstrated a change that is clinically relevant.

**Figure 2.**
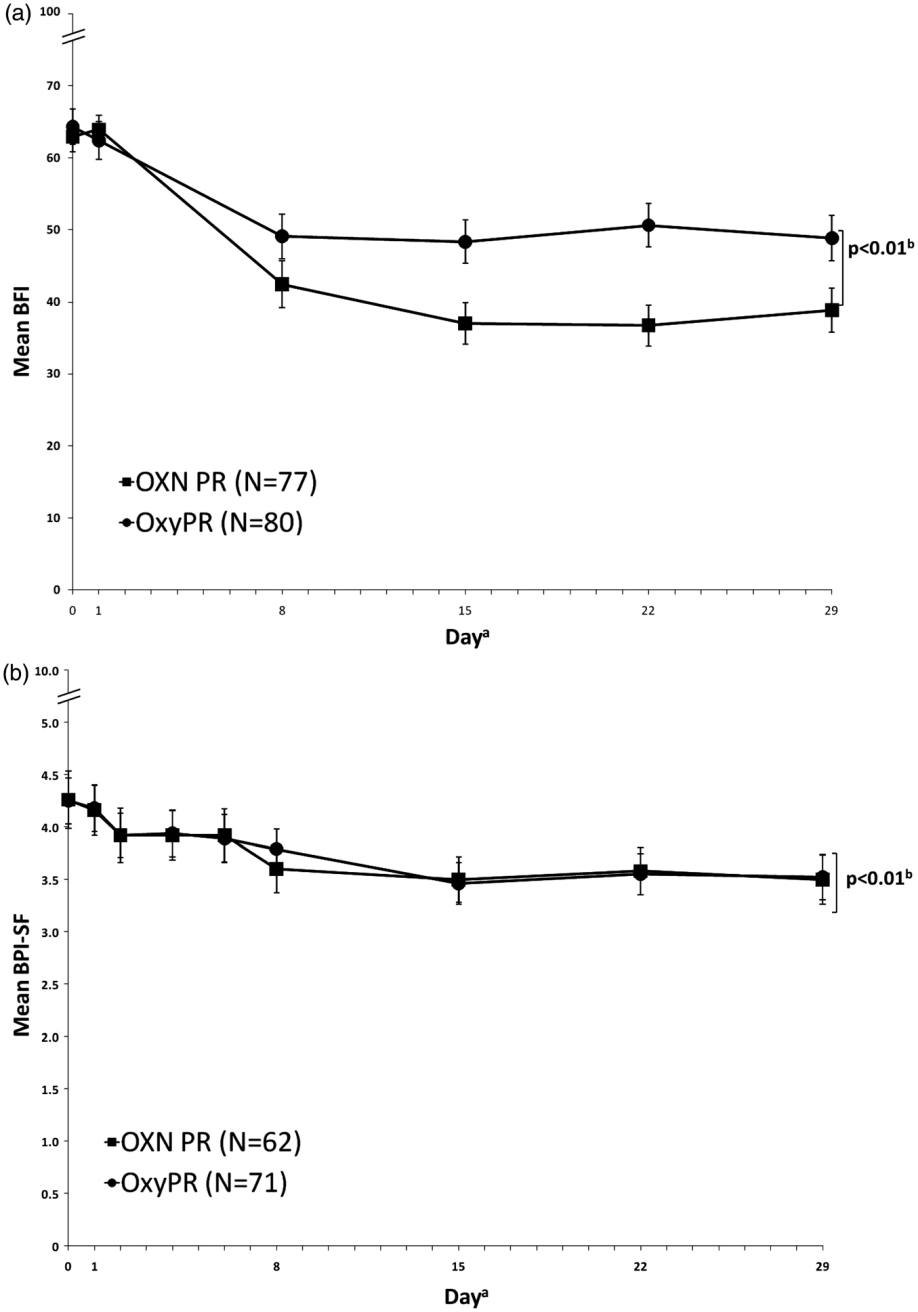
Effect of OXN PR and OxyPR treatment on (a) mean (± standard error) BFI score (full analysis population II, LOCF); and (b) mean (± standard error) BPI-SF score (per-protocol population, LOCF), by study day. ^a^Populations varied at each day. Error bars represent standard error; OXN ^b^*p*-value and confidence interval for treatment difference adjusted for baseline using an ANCOVA model. (a) BFI: 95% CI −19.03 to −3.24; (b) BPI-SF: 90% CI −0.47 to −0.45. OXN PR: oxycodone/naloxone prolonged-release tablets, OxyPR: oxycodone prolonged-release tablets, BFI: bowel function index. (Copyright for the BFI is owned by Mundipharma Research, 2002; the BFI is the subject of European Patent Application Publication No. EP 1 860 988 and corresponding patents and applications in other countries).

At randomization, mean (SD) BPI-SF scores were comparable for OXN PR and OxyPR treatment groups (4.16 [1.87] vs. 4.18 [1.87]). There was a slight decrease in mean BPI-SF scores, which was similar between groups. After 4 weeks of treatment, mean (SD) BPI-SF scores remained comparable between OXN PR and OxyPR groups (3.50 [1.88]) and 3.52 [1.80]; Figure 2B). Results of the primary analysis confirmed non-inferiority of OXN PR to OxyPR (LOCF, least squares [LS] mean difference −0.011; 90% CI: −0.47 to 0.45, *p* < 0.01). Non-inferiority in pain was further supported by sensitivity analyses, including MMRM, LOCF and BOCF.

#### Secondary endpoints

After 4 weeks, mean (SD) total laxative (oral bisacodyl) intake was 20% lower in the OXN PR group than the OxyPR group (26.10 [27.60] vs. 32.69 [31.26] mg, respectively), but the difference was not statistically significant (*p* = 0.17). The need for rescue analgesic medication was generally low in both treatment groups throughout the double-blind phase, in terms of both frequency (less than one intake/day) and dose. Differences between groups were not significant for either variable (*p* = 0.4 and *p* = 0.22, respectively). Over 4 weeks of treatment, PAC-SYM scores improved in both groups, but the degree of improvement at endpoint was significantly greater for OXN PR than for OxyPR, in terms of total symptom score (*p* = 0.014) and frequency of symptoms (*p* < 0.01; Table 2). These results are in line with results of BFI analyses, confirming better bowel efficacy of OXN PR, compared with OxyPR.

**Table 2.** Patient assessment of constipation using PAC-SYM (Full-Analysis II population)

Parameter	OXN PR, *n* = 77	OxyPR, *n* = 80
Total symptoms score		
Day 1		
*n*	75	80
Mean (SD)	17.38 (6.99)	18.21 (8.28)
Median (range)	17.0 (2–32)	16.0 (0–42)
At 4 weeks		
*n*	73	74
Mean (SD)	10.37 (8.57)	15.47 (9.83)
Median (range)	8.0 (0–37)	14.0 (0–37)
OXN PR versus OxyPR	*p* < 0.01	
Frequency of symptoms		
Day 1		
*n*	75	79
Mean (SD)	2.53 (1.09)	2.33 (1.07)
Median (range)	3.0 (0–4)	3.0 (0–4)
At 4 weeks		
*n*	73	73
Mean (SD)	1.47 (1.07)	2.03 (1.29)
Median (range)	1.0 (0–4)	2.0 (0–4)
OXN PR versus OxyPR	*p* < 0.01	

PAC-SYM: Patient Assessment of Constipation Symptoms, SD: standard deviation, OXN PR: oxycodone/naloxone prolonged-release tablets, OxyPR: oxycodone prolonged-release tablets.

At screening, mean EQ-5D index scores were comparable between treatment groups. After 4 weeks, mean (SD) index score was 0.50 (0.33) for OXN PR and 0.49 (0.38) for OxyPR. All mean (SD) EORTC QLQ-C30 scores were comparable between treatment groups at randomization. Mean pain subscore improved in both groups. Importantly, the mean (SD) constipation subscore in the OXN PR group was 44.32 (35.16) after 4 weeks of treatment (mean change from screening, −30.4), compared with a subscore of 59.78 (35.13) in the OxyPR group (mean change, −13.1). The improvement in this quality of life measure correlates well with the improvement seen measured by the BFI (R = 0.71)

### Safety

The proportion of patients who experienced AEs or ADRs was generally similar for OXN PR and OxyPR groups (Table 3). Approximately 20% of AEs were due to progression of underlying cancer disease. The most frequently reported AEs were GI disorders and ‘general disorders and administration site conditions’. The proportions of each group with ADRs were comparable for OXN PR and OxyPR (38.0 vs. 34.8%, respectively).

**Table 3.** Summary of adverse events, including most commonly affected (≥10% in any group) system organ classes (double-blind safety population)

Adverse events/organ classes	OXN PR, *n* = 92	OxyPR, *n* = 92
*Summary of adverse events*		
Total AEs		
Total number, *n*	270	243
Patients reporting, *n* (%)	79 (85.9)	71 (77.2)
AEs related to study medication* (ADR)		
Total number, *n*	77	62
Patients reporting, *n* (%)	35 (38.0)	32 (34.8)
Serious AEs related to study medication* (serious ADR)		
Total number, *n*	8	4
Patients reporting, *n* (%)	5 (5.4)	3 (3.3)
*Most commonly affected SOC*		
Gastrointestinal disorders, *n* (%)	34 (37.0)	28 (30.4)
Abdominal pain	7 (7.6)	5 (5.4)
Nausea	7 (7.6)	12 (13.0)
Vomiting	6 (6.5)	5 (5.4)
Diarrhoea	4 (4.3)	4 (4.3)
Worsened constipation	6 (6.5)	6 (6.5)
General disorders and administration site conditions, *n* (%)	27 (29.3)	27 (29.3)
Investigations, *n* (%)	25 (27.2)	25 (27.2)
Metabolism and nutrition disorders, *n* (%)	15 (16.3)	12 (13.0)
Nervous system disorders, *n* (%)	15 (16.3)	14 (15.2)
Neoplasm, *n* (%)	17 (18.5)	22 (23.9)

* As assessed by the investigator.

OXN PR: oxycodone/naloxone prolonged-release tablets, OxyPR: oxycodone prolonged-release tablets, AE: adverse event, ADR: adverse drug reaction, SOC: system organ class.

The incidence of serious ADRs was low in both groups (Table 3). In total, 18 patients (9.8%) died during the study, with nine in each group. No death was considered related to study medication (progression of malignant disease *n* = 16; cardiac disorders *n* = 2). There were no clinically important changes in vital signs, laboratory values, ECG assessments or modified SOWS observed. Mean (SD) SOWS values at baseline were 8.01 (7.76) and 8.90 (7.81) for OXN PR and OxyPR, respectively. These remained stable throughout the assessment period (OXN PR 6.64 [5.97]; OxyPR 7.29 [4.59] at last assessment).

## Discussion

Opioids are widely recommended to relieve pain in cancer patients,^3,4,31,45^ but are associated with AEs such as constipation. Results from this study demonstrate that OXN PR provides comparable analgesia to OxyPR for patients with moderate/severe cancer pain, whilst significantly improving bowel function and reducing symptoms of constipation.

OXN PR was superior to OxyPR with respect to bowel function, particularly reducing constipation, as measured by the BFI. Importantly, the difference between the two treatments was both statistically significant and clinically relevant and, moreover, achieved with less laxative use with OXN PR (20% less). In fact, there was already a statistically significant benefit in BFI score for OXN PR, compared with OxyPR, after 1 week. Overall, achieving this improvement in BFI with reduced laxative use in the OXN PR group, in such a short time frame, is encouraging, considering the severity of underlying disease and the negative impact of constipation on the lives of cancer patients.^9,10^ Primary efficacy findings were underlined by significant differences in PAC-SYM domain scores in favour of OXN PR.

With respect to the co-primary endpoint of pain relief, OXN PR was non-inferior to OxyPR, as measured by BPI-SF. Rescue analgesic use was low and comparable between groups throughout the double-blind phase, showing that patients were effectively titrated to a stable dose of study medication, and that comparable analgesic efficacy of OXN PR and OxyPR was not influenced by differential use of rescue analgesic medication.

General QoL results using EQ-5D and EORTC QLQ-C30 were similar for OXN PR and OxyPR treatments over time. However, results of the specific EORTC QLQ-C30 constipation subscore indicated that OXN PR patients had a superior outcome, compared with OxyPR patients. This could contribute towards an improved quality of life for many patients with chronic cancer pain treated with OXN PR.

There were slightly more dropouts due to AEs in the OXN PR group, although the overall incidence of AEs was comparable between treatment groups. Most frequently reported ADRs were consistent with the known safety profile of the opioid analgesic class of drugs, and with those seen in previous Phase III trials of OXN PR in patients with non-cancer pain.^26,27^ There were slightly more total GI disorders observed in the OXN PR group; however, the incidence of the most frequent AEs such as abdominal pain, worsened constipation, diarrhoea and vomiting were comparable, whilst nausea was reported less commonly in the OXN PR group. Therefore, the slightly higher number of total GI disorders in the OXN PR group is explained by the sum of less frequent AEs. It is possible that GI-related AEs indicate return of more active bowel function (as is aimed for when treating constipated patients), which has also been documented after use of other peripherally acting opioid antagonists, such as methylnaltrexone.^46^ In a study comparing methylnaltrexone with placebo, in patients receiving the active drug, abdominal pain also occurred more frequently. In addition, more flatulence and nausea were reported for methylnaltrexone;^46^ this was not seen with OXN PR in the study described here. There were no clinically relevant changes in laboratory values or vital signs related to study medication. Mean modified SOWS values were low and comparable in both groups. In summary, the results suggest that switching from other opioids to oxycodone – as either OXN PR or OxyPR – was generally safe and well tolerated by patients in this trial, with switching to OXN PR giving the added benefit of reduced OIC complications.

The findings of this study should be interpreted within the context of the trial design and patient population. The double-blind phase had a duration of 4 weeks to allow for assessment of analgesic effect, consistent with European Medicines Agency guidelines on clinical studies of chronic moderate/severe cancer pain.^47^ This timeframe is also deemed adequate for observing differences in constipation based on previous Phase II^48^ and Phase III data.^27^

Although the demographic profile of both treatment groups was generally similar there was a slightly higher percentage of patients aged ≤ 65 years in the OXN PR group than in the OxyPR treatment group. However, this difference is not clinically relevant, as results from previous clinical studies have demonstrated that the efficacy and safety of OXN PR in older patients is similar to that in younger patients.^49^ Additional assessments showed that there was no correlation between BFI score at study end (LOCF) and age (data not shown).

Opioid-induced bowel dysfunction is common and adds to the burden of living with chronic pain. Current oral and rectal laxatives are often ineffective and do not have a good evidence base.^15^ The new targeted approach of administering peripherally acting opioid antagonists is effective and is supported by extensive clinical trial data. Previous attempts to use immediate-release naloxone were unsuccessful because of reversal of analgesia; and the only other licensed peripherally acting opioid antagonist, methylnaltrexone, has to be given by injection.^15,46^ The present trial has shown that oral OXN PR tablets are well tolerated and can effectively and conveniently provide targeted treatment of OIC at a dose range which includes a substantial proportion of patients with cancer pain.

## Conclusion

In this study of patients with OIC and moderate/severe cancer pain, patients who were switched directly from other opioids to OXN PR experienced a similar analgesic effect as well as a statistically significant and clinically relevant improvement in bowel function, compared with those switched to OxyPR. Overall, these data are in line with results of previous Phase III trials demonstrating analgesic efficacy and tolerability of OXN PR, suggesting that the combination of oxycodone and naloxone is suitable across the spectrum of patients with cancer and non-cancer pain.

## Clinical trials number

ClinicalTrials.gov identifier: NCT00513656

EudraCT Number: 2007-001313-42
